# Management of splenomegaly in patients with myelofibrosis in the era of JAK inhibitors: a comprehensive review

**DOI:** 10.3389/fonc.2026.1851781

**Published:** 2026-05-20

**Authors:** Yousef Al-Asa’d, Nabeel Qasem, Awni Alshurafa, Khalil Al-Farsi, Mohamed A. Yassin

**Affiliations:** 1 Department of Hematology, National Center for Cancer Care and Research (NCCCR), Hamad Medical Corporation, Doha, Qatar; 2 Hematology Department, National Hematology and Bone Marrow Transplant Center, University Medical City, Muscat, Oman; 3 College of Medicine, Qatar University, Doha, Qatar

**Keywords:** combination therapy, splenomegaly, fedratinib, JAK inhibitors, momelotinib, myelofibrosis, pacritinib, ruxolitinib

## Abstract

Myelofibrosis (MF) is a Philadelphia chromosome–negative myeloproliferative neoplasm characterized by progressive bone marrow fibrosis, constitutional symptoms, cytopenias, and splenomegaly. Splenic enlargement, driven primarily by extramedullary hematopoiesis, represents a hallmark of MF and contributes substantially to symptom burden, portal hypertension, and worsening cytopenias through splenic sequestration. The identification of constitutive Janus kinase–signal transducer and activator of transcription (JAK–STAT) pathway activation as the central pathogenic mechanism in myeloproliferative neoplasms led to the development of JAK inhibitors, which have become the cornerstone of therapy for spleen volume reduction and symptom amelioration. Four JAK inhibitors—ruxolitinib, fedratinib, pacritinib, and momelotinib—are currently approved by regulatory agencies, each exhibiting distinct pharmacological profiles suited to different clinical phenotypes. Ruxolitinib remains the first-line standard for patients with adequate platelet counts, pacritinib is preferred in severe thrombocytopenia, and momelotinib is the agent of choice in anemia-dominant disease. Fedratinib serves as an effective second-line option following ruxolitinib failure. Emerging combination strategies, including pelabresib plus ruxolitinib and navitoclax plus ruxolitinib, have demonstrated superior spleen volume reduction in phase III trials and represent a promising therapeutic frontier. Conventional agents such as hydroxyurea and immunomodulatory drugs retain a role in select patients. Non-pharmacologic interventions—including splenectomy, splenic irradiation, partial splenic artery embolization, and radiofrequency ablation—remain valuable for patients refractory to or ineligible for medical therapy. This review provides a comprehensive and updated overview of the management of splenomegaly in MF, integrating all four approved JAK inhibitors, emerging combination therapies, peri-transplant considerations, and procedural approaches, and proposes a practical phenotype-driven treatment framework.

## 1 Introduction

Philadelphia chromosome–negative myeloproliferative neoplasms (MPNs) encompass a group of clonal hematopoietic stem cell disorders, the three classical entities being polycythemia vera (PV), essential thrombocythemia (ET), and myelofibrosis (MF). These disorders share overlapping pathophysiology and clinical features, with both PV and ET carrying the potential to evolve into secondary MF over time (1, 2). All MPNs harbor an inherent risk of leukemic transformation to acute myeloid leukemia (AML), which is associated with a dismal prognosis (3).

MPNs originate from a somatically mutated hematopoietic stem cell (HSC) that undergoes clonal expansion, giving rise to the majority of myeloid cells as well as B and natural killer (NK) lymphocytes. This clonal proliferation is accompanied by single or multilineage hyperplasia (4). Three principal driver mutations have been identified as the molecular underpinnings of MPN pathogenesis: *JAK2*, *MPL*, and *CALR*. The *JAK2* V617F mutation is the most prevalent, occurring in approximately 95% of PV cases and 50–60% of ET and primary MF (PMF) patients (4). These driver mutations converge on the constitutive activation of JAK2 kinase–dependent cytokine receptor signaling, particularly the JAK/STAT pathway, which leads to transcriptional dysregulation and promotes clonal expansion (4). Notably, even “triple-negative” MPN cases that lack all three canonical driver mutations demonstrate upregulation of JAK/STAT target genes, affirming the central role of this pathway in disease pathogenesis (5). This universal dependence on hyperactive JAK/STAT signaling provides the biological rationale for the therapeutic efficacy of JAK inhibitors in MF, irrespective of the underlying driver mutation status (1).

The clinical presentation of classical BCR::ABL1-negative MPNs spans a broad spectrum, from asymptomatic disease detected incidentally to constitutional symptoms, thrombotic and hemorrhagic events, and splenomegaly-related complaints. PV is primarily characterized by erythrocytosis, often accompanied by leukocytosis or thrombocytosis, and carries a heightened risk of vascular events. ET is defined by persistent thrombocytosis with the presence of enlarged, mature, hyperlobated megakaryocytes in the bone marrow. PMF involves dysplastic megakaryocyte and granulocyte proliferation, progressing through a prefibrotic/early stage with hypercellular marrow and minimal reticulin fibrosis to overt fibrotic-stage PMF characterized by advanced fibrosis, leukoerythroblastosis, anemia, splenomegaly, and hepatomegaly (6).

Splenomegaly is among the most clinically significant manifestations of MF and is directly attributable to splenic extramedullary hematopoiesis (EMH). Progressive splenic enlargement is associated with debilitating symptoms including early satiety, abdominal discomfort, portal hypertension, diminished functional capacity, and worsening cytopenias secondary to splenic sequestration (7). Massive splenomegaly is particularly prevalent in MF, with approximately 38% of patients presenting with a palpable spleen extending at least 10 cm below the left costal margin (1). The definition of massive splenomegaly is not uniformly standardized across clinical studies or guidelines. However, in clinical practice it is most defined as a palpable spleen extending more than 10 cm below the left costal margin — a threshold applied in both observational and interventional MF studies. However, cut-offs reported in the literature range from 5 cm to more than 10 cm below the costal margin, limiting cross-study comparisons of splenomegaly severity, and palpation can have a significant inter-observer variability. Nevertheless, palpable spleen size retains practical utility and has been formally incorporated into established prognostic scoring systems. The JAK–STAT pathway promotes CXCL12-dependent chemotaxis and EMH; accordingly, PMF patients harboring homozygous *JAK2* V617F mutations tend to exhibit larger spleens (p = 0.003) and higher white blood cell counts compared to those with heterozygous or wild-type alleles (8). Conversely, patients with *CALR* mutations have been observed to have longer splenomegaly-free survival intervals (9).

A seminal review by Tremblay et al. published in 2020 provided a comprehensive framework for splenomegaly management that included ruxolitinib and fedratinib—the only two approved JAK inhibitors at that time—along with splenectomy, splenic irradiation, and partial splenic artery embolization (1). Since that publication, two additional JAK inhibitors—pacritinib (approved February 2022) and momelotinib (approved September 2023)—have received regulatory approval, landmark phase III combination therapy data have emerged, and the understanding of peri-transplant JAK inhibitor use has matured substantially. The present review builds upon and updates that foundation by incorporating all four approved JAK inhibitors with a practical phenotype-driven selection framework, emerging combination therapies, peri-transplant considerations, and a comprehensive evaluation of non-pharmacologic approaches including radiofrequency ablation.

## 2 JAK inhibitors

The identification of the *JAK2* V617F mutation and the subsequent recognition of constitutive JAK–STAT pathway activation as the central driver of MPN pathogenesis revolutionized the therapeutic landscape of MF (4). JAK inhibitors have since become the cornerstone of therapy for managing splenomegaly and alleviating symptom burden. Four agents are currently approved by regulatory bodies: ruxolitinib, fedratinib, pacritinib, and momelotinib (10, 11). Each exhibits a distinct kinase inhibition profile and clinical niche, and together they provide a versatile pharmacologic toolkit for managing splenomegaly across the spectrum of MF presentations (**Table 1**).

**Table 1:** Comparison of approved JAK inhibitors for myelofibrosis-associated splenomegaly.

Agent (targets)	Key trials	Spleen efficacy (SVR35)	Symptom efficacy	Cytopenias profile	Key non-hematologic toxicities	Best-fit phenotype
Ruxolitinib (JAK1/JAK2)	COMFORT-I (vs placebo) COMFORT-II (vs BAT)	~42% at wk 24 (COMFORT-I) ~28% at wk 48 (COMFORT-II) Rapid onset; durable in responders	Strong symptom benefit vs placebo/BAT	Common: dose-limiting anemia + thrombocytopenia Platelet-based dosing (≥50×10^9^/L required)	Infections (herpes zoster) Weight gain/edema Discontinuation syndrome – taper required NMSC Increase in LDL	First-line standard when platelets adequate and cytopenias manageable
Fedratinib (JAK2/FLT3)	JAKARTA (frontline vs placebo) JAKARTA-2 (post-ruxolitinib)	~47% at wk 24 (JAKARTA, 400 mg) Meaningful activity post-ruxolitinib (JAKARTA-2: ~55% by revised criteria)	Symptom improvement demonstrated in pivotal studies	Common: anemia + thrombocytopenia Can limit use if baseline cytopenic	GI toxicity (nausea, vomiting, diarrhea) Wernicke encephalopathy risk – monitor thiamine	Second-line after ruxolitinib if platelets adequate; also first-line option
Pacritinib (JAK2/FLT3/IRAK1)	PERSIST-1 (vs BAT) PERSIST-2 (thrombocytopenic MF; BAT incl. ruxolitinib)	~19% overall (PERSIST-1) ~29% vs 3% BAT (PERSIST-2, 200 mg BID) Notable benefit in plt <50×10^9^/L	Symptom benefit seen in trials, including cytopenic populations	Relatively less myelosuppressive JAK1-sparing; designed for thrombocytopenia	GI toxicity (diarrhea, nausea) Monitor for bleeding and cardiac events (historical safety signal)	Preferred when platelets <50×10^9^/L (severe thrombocytopenia)
Momelotinib (JAK1/JAK2 + ACVR1)	SIMPLIFY-1 (vs ruxolitinib) SIMPLIFY-2 (vs BAT) MOMENTUM (vs danazol, anemic post-JAKi)	~26.5% (non-inferior to ruxolitinib, SIMPLIFY-1) Lower SVR35 post-ruxolitinib (SIMPLIFY-2)	Variable vs ruxolitinib (SIMPLIFY-1: did not meet non-inferiority for TSS) MOMENTUM: superior vs danazol in anemic/post-JAKi population	Anemia advantage: ACVR1 → ↓hepcidin → improved iron availability Improves transfusion independence vs comparators Thrombocytopenia still occurs but less limiting for erythropoiesis	Peripheral neuropathy (may be irreversible) GI toxicity (less prominent) Monitor anemia/transfusion metrics Cardiac toxicity	Preferred when anemia/transfusion dependence is central, either frontline or after prior JAKi

BAT, best available therapy; BID, twice daily; GI, gastrointestinal; JAKi, JAK inhibitor; LDL, low-density lipoprotein; MF, myelofibrosis; MFSAF, Myelofibrosis Symptom Assessment Form; NMSC, non-melanoma skin cancers; plt, platelets; SVR35, ≥35% spleen volume reduction; TSS, Total Symptom Score; wk, week.

### 2.1 Ruxolitinib

Ruxolitinib was the first JAK inhibitor to receive regulatory approval for MF and functions as a potent inhibitor of both JAK1 and JAK2 (10, 12). Its dual-kinase inhibition mediates anti-inflammatory effects through JAK1 suppression, which accounts for its symptom-relieving properties, while JAK2 inhibition targets the neoplastic proliferative drive responsible for organomegaly (11).

The pivotal COMFORT-I and COMFORT-II trials established ruxolitinib as the standard of care. In COMFORT-I, 41.9% of patients achieved a spleen volume reduction of at least 35% (SVR35) at week 24, compared to 0.7% in the placebo group (13). COMFORT-II demonstrated that 28% of ruxolitinib-treated patients achieved SVR35 at week 48 versus none in the best available therapy (BAT) arm (14). Long-term follow-up data have suggested a potential survival advantage with ruxolitinib, attributed in part to the reduction of catabolic stress and physical debility associated with massive splenomegaly (13, 14).

The principal adverse effects of ruxolitinib are hematologic, specifically dose-dependent anemia and thrombocytopenia, which frequently manifest early in treatment and may necessitate dose modifications (11). Platelet-based dosing is employed, with a typical requirement of platelets ≥50 × 10^9^/L for initiation. This usually refers to a starting dose of 10 mg twice daily, however, this dosing is associated with attenuated spleen volume reduction and inferior symptom control compared to the standard doses of 15–20 mg BD established in the COMFORT trials (12–14). Clinicians should aim to up titrate the dose as tolerated, targeting the highest dose that maintains an acceptable platelet nadir or consider switching to another agent. Non-hematologic toxicities include increased susceptibility to opportunistic infections—particularly herpes zoster reactivation—and weight gain (11, 15). Ruxolitinib has also been associated with an increased incidence of non-melanoma skin cancers (NMSC), particularly squamous cell carcinoma (SCC) and basal cell carcinoma (BCC). A 10-year retrospective cohort study demonstrated that ruxolitinib-exposed patients with MPN had an adjusted NMSC hazard ratio of 2.69, with an even higher SCC risk (HR 3.24) (16). These findings are consistent with the long-term COMFORT-I follow-up data, in which NMSC was identified in 17.1% of ruxolitinib-treated patients versus 2.1% on best available therapy (13). Periodic dermatologic assessment is therefore recommended for all patients receiving long-term ruxolitinib. Additionally, ruxolitinib treatment is associated with clinically meaningful increases in total cholesterol and low-density lipoprotein (LDL) cholesterol, as demonstrated in a *post-hoc* metabolic analysis of COMFORT-I (17).

A clinically important phenomenon is ruxolitinib discontinuation syndrome, characterized by rapid symptom recurrence, acute splenomegaly rebound, and potential hemodynamic instability upon abrupt cessation; gradual tapering is therefore essential when discontinuation is required (13).

### 2.2 Fedratinib

Fedratinib is a selective JAK2 inhibitor with additional activity against FLT3, approved both as a first-line agent and as a second-line option for patients who are resistant or intolerant to ruxolitinib (10, 18). Its relative selectivity for JAK2 over JAK1 distinguishes it pharmacologically from ruxolitinib, potentially conferring a different therapeutic and toxicity profile (11).

The JAKARTA trial evaluated fedratinib in JAK inhibitor–naïve patients and demonstrated that 47% of patients receiving the 400 mg dose achieved SVR35 at week 24, significantly exceeding the placebo group (18, 19). The JAKARTA-2 trial, which enrolled patients previously treated with ruxolitinib, reported an SVR35 of 55% in the original per-protocol analysis using last-observation-carried-forward methodology; a subsequent intention-to-treat reanalysis applying stringent criteria for ruxolitinib failure demonstrated an SVR35 of 31%, establishing fedratinib as a valuable salvage option for splenomegaly following first-line failure (20).

The safety profile of fedratinib is notable for gastrointestinal toxicity, including nausea, vomiting, and diarrhea, which are common particularly during the early treatment period but are generally manageable with supportive care (11). A unique and serious concern is the risk of Wernicke encephalopathy secondary to thiamine deficiency. Although rare, this potential complication led to a clinical hold during development; consequently, thiamine levels must be assessed before and periodically during treatment, and supplementation is often recommended (19).

### 2.3 Pacritinib

Pacritinib is a macrocyclic JAK2 inhibitor with additional activity against IRAK1 and FLT3 and minimal inhibition of JAK1 (11). This “JAK1-sparing” property is hypothesized to preserve interleukin-6 (IL-6) signaling, which plays a role in megakaryopoiesis, making pacritinib particularly suited for patients with severe thrombocytopenia who cannot tolerate therapeutic doses of other JAK inhibitors (11).

The PERSIST-1 and PERSIST-2 trials established the efficacy of pacritinib (21, 22). PERSIST-2 specifically enrolled patients with platelet counts ≤100 × 10^9^/L and compared pacritinib to BAT, which included ruxolitinib. The trial demonstrated that pacritinib at 200 mg twice daily was significantly more effective than BAT in achieving SVR35 (29% vs. 3%) (22). Crucially, pacritinib maintained its efficacy even in patients with platelets below 50 × 10^9^/L—a population often ineligible for therapeutic doses of ruxolitinib (22).

The principal adverse effects include gastrointestinal disturbances (diarrhea, nausea) and anemia (11). Early clinical development was complicated by safety signals regarding hemorrhagic and cardiac events, prompting dose optimization studies. The currently approved dose of 200 mg twice daily represents the optimal balance between efficacy and safety in the cytopenic population (22).

### 2.4 Momelotinib

Momelotinib is a JAK1 and JAK2 inhibitor that uniquely also inhibits activin A receptor type 1 (ACVR1/ALK2) (23). Inhibition of ACVR1 reduces hepatic hepcidin production, thereby increasing iron availability for erythropoiesis (11). This dual mechanism addresses the anemia of chronic inflammation that frequently complicates MF, distinguishing momelotinib from the other approved agents (10).

The SIMPLIFY-1 trial compared momelotinib to ruxolitinib in JAK inhibitor–naïve patients, demonstrating non-inferiority for SVR35 at week 24 (26.5% vs. 29%) but superior rates of transfusion independence (10, 24). However, momelotinib did not achieve non-inferiority for the Total Symptom Score (TSS) response, with ruxolitinib demonstrating superior symptom reduction (24). An important limitation of SIMPLIFY-1 that requires acknowledgment: the ruxolitinib comparator arm was capped at a maximum dose of 10 mg twice daily — substantially below the 15–20 mg twice daily doses employed in the pivotal COMFORT-I and COMFORT-II trials (12–14). This dose restriction reflected the cytopenic patient population enrolled in SIMPLIFY-1 (22), but directly resulted in attenuated ruxolitinib response rates compared to those observed in the COMFORT program.

The MOMENTUM trial further solidified momelotinib’s role in symptomatic, anemic patients previously treated with a JAK inhibitor, showing superior symptom and spleen responses compared to danazol (25). These data position momelotinib as the agent of choice when anemia and transfusion dependence are the dominant clinical features alongside splenomegaly.

Adverse effects include peripheral neuropathy (10, 11). which is tends to be most prominent during the first six months of treatment; close neurological assessment is therefore especially important during this early period, and prompt dose modification or discontinuation should be considered if symptoms emerge or worsen (24–26). A cardiac safety signal has been reported with momelotinib in clinical trial data and in integrated long-term safety analyses; baseline cardiovascular evaluation with periodic cardiac monitoring during therapy is therefore advisable (26). Gastrointestinal toxicity and thrombocytopenia may also occur, although momelotinib is generally less myelosuppressive with respect to erythropoiesis compared to ruxolitinib (25). With respect to thrombocytopenia, momelotinib may be initiated and maintained in patients with severe thrombocytopenia (platelets <50 × 10^9^/L), however, dose interruption is generally recommended when platelet counts fall below 20 × 10^9^/L to mitigate hemorrhagic risk (25).

## 3 Practical selection and switching strategy

### 3.1 Selection of first-line agent

Choosing the appropriate JAK inhibitor requires a personalized approach based on the patient’s baseline blood counts, symptom profile, and comorbidities.

**Figure 1:**
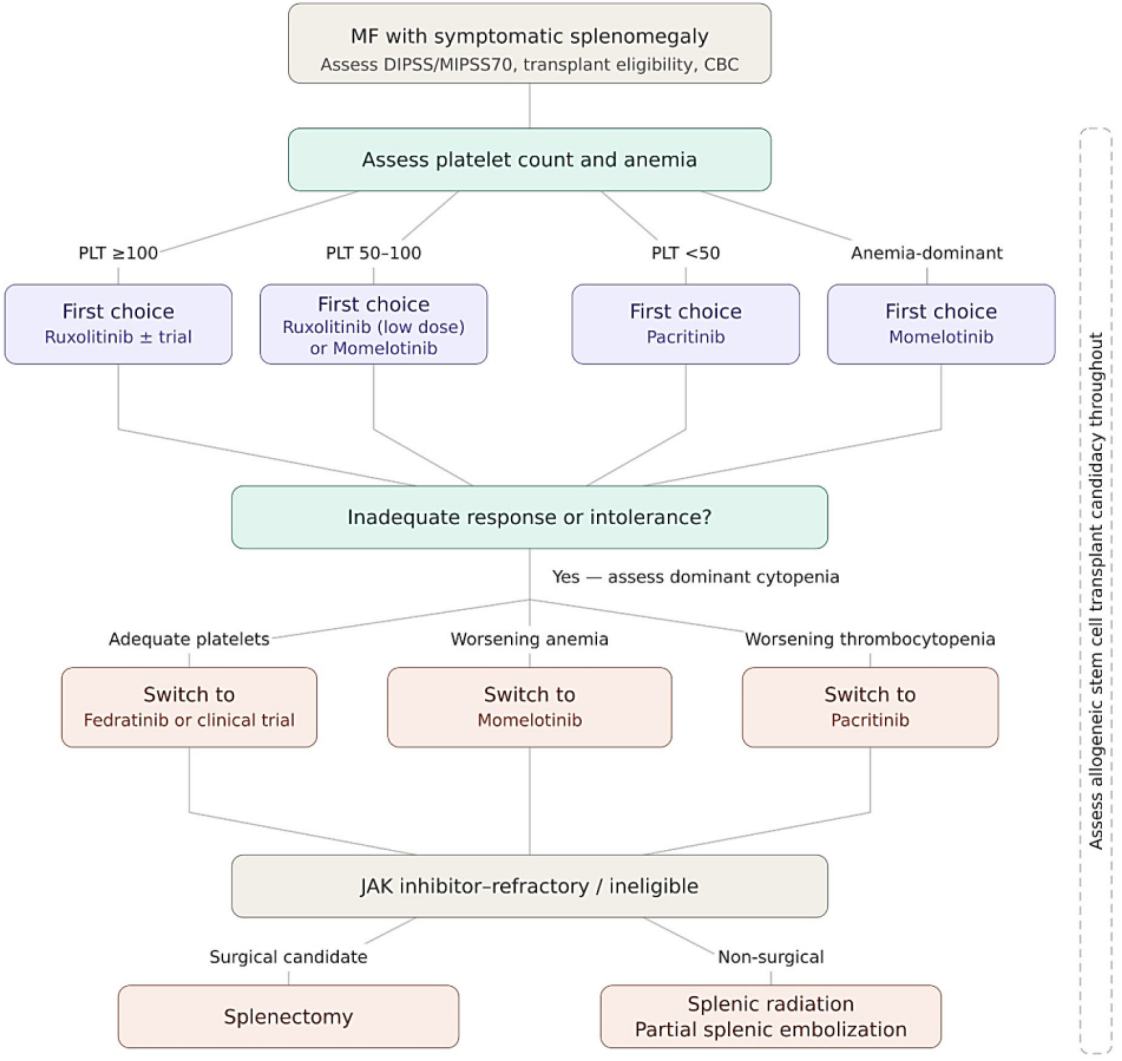
Proposed phenotype-driven algorithm for the management of splenomegaly in myelofibrosis. The selection of JAK inhibitor therapy is guided by the patient’s baseline platelet count, anemia status, transfusion dependence, and prior treatment history. Low-dose ruxolitinib refers to a starting dose of 10 mg BD.

For patients with standard-risk blood counts (platelets >100 × 10^9^/L), ruxolitinib remains the standard of care owing to its extensive long-term safety data and robust efficacy in both spleen and symptom control (13, 14). In patients presenting with severe thrombocytopenia (platelets <50 × 10^9^/L), pacritinib is the preferred agent as it is the only inhibitor specifically approved for this population and can be administered at full dose without exacerbating thrombocytopenia (22). For patients with an anemia-dominant phenotype, momelotinib is the optimal choice, particularly in those who are transfusion-dependent, as it addresses the underlying iron metabolism dysregulation through ACVR1 inhibition (25). Following ruxolitinib failure, fedratinib represents a strong second-line candidate for patients with preserved platelet counts who require aggressive spleen reduction (19, 20). A proposed phenotype-driven algorithm for JAK inhibitor selection is presented in **Figure 1**.

### 3.2 Switching between JAK inhibitors

Transitioning between JAK inhibitors requires careful management to avoid “cytokine rebound”—a phenomenon in which symptoms flare rapidly upon cessation of JAK inhibition due to the surge of previously suppressed inflammatory cytokines (10, 11, 27).

For the ruxolitinib-to-momelotinib transition, a direct switch is generally safe owing to momelotinib’s JAK1 inhibitory activity, which provides continued cytokine suppression. Patients can discontinue ruxolitinib and initiate momelotinib the following day without a washout period (10, 27). In contrast, the ruxolitinib-to-fedratinib transition requires greater caution because fedratinib lacks significant JAK1 inhibition. Abrupt ruxolitinib cessation in this setting may trigger a cytokine storm. A practical approach involves tapering ruxolitinib over 1–2 weeks while initiating fedratinib, or employing a brief overlap period. Alternatively, a strict washout may be used with careful monitoring and corticosteroid coverage for rebound symptoms (11).

## 4 Head-to-head comparisons

Direct comparative data among JAK inhibitors remain limited. The majority of evidence regarding relative efficacy is derived from indirect treatment comparisons (ITCs), network meta-analyses (NMAs), and distinct patient stratifications based on cytopenias.

The only phase III trial directly comparing two JAK inhibitors in treatment-naïve patients is the SIMPLIFY-1 trial, which randomized patients to momelotinib or ruxolitinib. Regarding splenic efficacy, momelotinib met the primary endpoint of non-inferiority to ruxolitinib, with SVR35 at week 24 achieved by 26.5% of the momelotinib group versus 29% of the ruxolitinib group (with maximum dose of 10 mg twice daily) (24). However, momelotinib failed to demonstrate non-inferiority for TSS response; ruxolitinib showed superior symptom reduction (42% vs. 28% achieving ≥50% TSS reduction). The principal advantage of momelotinib resided in its anemia profile, with significantly higher rates of transfusion independence compared to ruxolitinib (24).

In the absence of direct trials between ruxolitinib and fedratinib or pacritinib, clinicians must rely on indirect comparisons. A matching-adjusted indirect comparison (MAIC) of JAKARTA versus COMFORT-I data suggested comparable efficacy between fedratinib and ruxolitinib for both SVR35 and TSS50 when adjusting for baseline characteristics (28). A network meta-analysis by Agarwal et al. evaluating all four approved inhibitors found no statistically significant differences in SVR35 rates between ruxolitinib and fedratinib in the first-line setting (29). Pacritinib’s unique positioning for severely thrombocytopenic patients was reinforced by a retrospective analysis of PERSIST-2, in which pacritinib demonstrated superior SVR35 rates compared to ruxolitinib when used as BAT in patients with baseline thrombocytopenia (22).

Agent selection is thus often driven more by safety, tolerability, and the patient’s cytopenia profile than by differential splenic efficacy alone. Ruxolitinib and fedratinib commonly cause dose-dependent cytopenias that may necessitate dose reductions and blunt splenic control. Momelotinib and pacritinib are generally considered more cytopenia-sparing. Fedratinib carries higher rates of gastrointestinal toxicity and a boxed warning for Wernicke encephalopathy requiring thiamine monitoring (18). Ruxolitinib’s association with opportunistic infections, particularly herpes zoster, may extend across the JAK inhibitor class but is best documented for ruxolitinib due to longer real-world exposure (15).

To provide a structured overview of the relative efficacy profiles of the four approved JAK inhibitors, **Table 2** presents an indirect comparison across the domains of spleen volume response (SVR35), symptom control (TSS50), and anemia or transfusion independence. Several critical methodological caveats must accompany such a comparison (29): (1) the trials enrolled distinct patient populations with different baseline platelet counts, hemoglobin levels, and degrees of prior JAK inhibitor exposure; (2) comparators varied across programs (placebo, best available therapy, ruxolitinib at varying doses, and danazol); (3) endpoint definitions and reporting conventions were not uniform. Despite these limitations, clinically meaningful patterns emerge across the four agents (12–14, 16–20, 22, 23). Importantly, death rates during initial study periods do not appear to differ substantially among these agents, supporting the principle that patient-matched drug selection — optimized for cytopenia profile, symptom burden, and tolerability — may be more clinically consequential than differences in absolute SVR35 rates (29).

**Table 2:** Indirect comparison of approved JAK inhibitors for myelofibrosis: spleen response, symptom control, and anemia/transfusion independence.

Agent	Spleen response (SVR35)	Symptom response (TSS50)	Anemia / Transfusion independence	Key population & study caveats
Ruxolitinib *(JAK1/JAK2) COMFORT-I, COMFORT-II* (12–14)	~42% SVR35 at wk 24 (COMFORT-I) ~28% at wk 48 (COMFORT-II)	~46% TSS50 (COMFORT-I) Superior vs placebo/BAT	No specific anti-anemia indication Anemia + thrombocytopenia are dose-limiting TI not a primary endpoint	JAKi-naïve; plt ≥100×10^9^/L at standard dose; comparator = placebo or BAT; 15–20 mg BID (12–14)
Fedratinib *(JAK2/FLT3) JAKARTA, JAKARTA-2* (16–18)	~47% SVR35 at wk 24 (JAKARTA) ~31% by stringent ITT (JAKARTA-2)	~40% TSS50 (JAKARTA) Superior vs placebo	No anti-anemia mechanism Anemia + thrombocytopenia occur	Frontline (JAKARTA) and post-ruxolitinib (JAKARTA-2); plt ≥50×10^9^/L; GI toxicity + Wernicke risk (16–18)
Pacritinib *(JAK2/FLT3/IRAK1) PERSIST-1, PERSIST-2* (19, 20)	~29% vs 3% BAT (PERSIST-2, plt ≤100×10^9^/L) Efficacy maintained at plt <50×10^9^/L	~25% TSS50 (PERSIST-2)	JAK1-sparing: less erythropoietic suppression Thrombocytopenia relatively spared	Specifically thrombocytopenic MF; comparator included ruxolitinib as BAT; 200 mg BID (19, 20)
Momelotinib *(JAK1/JAK2/ACVR1) SIMPLIFY-1, MOMENTUM* (22, 23)	~26.5% (non-inferior to ruxolitinib, SIMPLIFY-1) Note: ruxolitinib capped at 10 mg BID in SIMPLIFY-1 (22)	~28% TSS50 (SIMPLIFY-1) — did NOT meet non-inferiority vs ruxolitinib (22) Superior vs danazol (MOMENTUM) (23)	~67% TI vs 49% ruxolitinib (SIMPLIFY-1) (22) ACVR1 inhibition → ↓hepcidin → improved iron availability (21) Best TI data across approved agents	SIMPLIFY-1: JAKi-naïve, ruxolitinib capped 10 mg BID (vs COMFORT 15–20 mg BID) — limits cross-trial comparison (22); MOMENTUM: anemic, post-JAKi (23)

These trials enrolled distinct patient populations (different baseline platelet counts, hemoglobin levels, and prior JAKi exposure). Comparators varied (placebo, BAT, ruxolitinib, danazol). Response endpoints were not uniformly reported. Importantly, death rates during initial study periods do not appear to differ substantially between agents, supporting the principle that drug selection should be driven primarily by patient phenotype, cytopenia profile, and tolerability rather than by cross-trial SVR35 comparisons alone (12–14, 16–20, 22, 23, 27).

BAT, best available therapy; BID, twice daily; JAKi, JAK inhibitor; MF, myelofibrosis; plt, platelets; SVR35, ≥35% spleen volume reduction; TI, transfusion independence; TSS50, ≥50% reduction in Total Symptom Score.

An important conceptual distinction must be emphasized: while the availability of pacritinib and momelotinib has extended JAK inhibitor therapy to patients with severe thrombocytopenia and anemia-dominant disease, respectively, none of the four approved JAK inhibitors carries a regulatory indication for the treatment or improvement of cytopenias per se (10). These agents are indicated for splenomegaly and disease-related symptom control in cytopenic patients who would otherwise be ineligible for therapy — not for the primary purpose of correcting thrombocytopenia or reversing anemia. In clinical practice, platelet counts often cannot be reliably raised to meaningful levels with any currently approved agent, and a substantial proportion of momelotinib-treated patients remain transfusion-dependent despite therapy (24, 25). Effective management of severe thrombocytopenia and transfusion-refractory anemia therefore continues to represent a critical unmet need in myelofibrosis, underscoring the urgency of novel therapeutic strategies specifically targeting these manifestations (22).

## 5 Emerging combination therapies

While JAK inhibitors represent the backbone of MF therapy, they do not eradicate the malignant clone and their effects are often not sustained long-term. The future of MF treatment lies in combination strategies targeting alternative pathways to enhance efficacy and achieve disease modification.

### 5.1 Pelabresib plus ruxolitinib

Pelabresib is a bromodomain and extra-terminal domain (BET) inhibitor that works synergistically with JAK inhibitors by downregulating NF-κB–driven inflammatory gene expression and reducing pro-inflammatory cytokine production. The phase III MANIFEST-2 trial compared ruxolitinib plus pelabresib versus ruxolitinib plus placebo in JAK inhibitor–naïve MF patients with splenomegaly and symptoms. The combination achieved SVR35 in approximately 66% of patients compared to 35% with ruxolitinib monotherapy, representing a near doubling of the spleen response rate (30). The combination was also associated with improvements in bone marrow fibrosis and was generally well tolerated without significant additive toxicity (30). These data represent one of the most significant therapeutic advances in MF since the approval of ruxolitinib (**Table 3**). Updated data from the phase 3 MANIFEST-2 trial presented at the 2024 American Society of Hematology (ASH) Annual Meeting provided important safety and efficacy context (31). An earlier analysis had raised concern regarding a numerical imbalance in leukemic transformation rates between the pelabresib–ruxolitinib arm (6.1%) and the placebo–ruxolitinib arm (4.2%); however, updated follow-up data demonstrated that this imbalance diminished over time, with the transformation rate in the combination arm falling within the range historically observed in MF populations, thereby supporting a favorable benefit–risk profile for the combination (31).

**Table 3:** Emerging combination therapies for myelofibrosis-associated splenomegaly (Phase III Data).

Combination	Trial	Population	SVR35 (combination vs control)	Key secondary endpoints	Clinical significance
Pelabresib + Ruxolitinib	MANIFEST-2 (Phase III, randomized, double-blind)	JAKi-naïve MF with splenomegaly	~66% vs ~35%	Improved bone marrow fibrosis; favorable cytokine modulation; well tolerated	Near doubling of spleen response rate; potential for disease modification through BET inhibition of NF-κB pathway
Navitoclax + Ruxolitinib - Discontinued	TRANSFORM-1 (Phase III, randomized, double-blind)	Untreated intermediate/high-risk MF	63.2% vs 31.5%	SVR35 at any time: 77% vs 42%; ongoing evaluation of bone marrow fibrosis and survival	Targets apoptotic resistance (BCL-xL/BCL-2); may overcome JAKi resistance in high-risk molecular subtypes

BET, bromodomain and extra-terminal domain; JAKi, JAK inhibitor; MF, myelofibrosis; SVR35, ≥35% spleen volume reduction.

### 5.2 Navitoclax plus ruxolitinib - discontinued

Navitoclax is a BCL-xL and BCL-2 inhibitor that targets the apoptotic pathway and has shown promise in overcoming JAK inhibitor resistance. The phase III TRANSFORM-1 trial evaluated the combination of ruxolitinib plus navitoclax versus ruxolitinib plus placebo in untreated intermediate- or high-risk MF. The combination demonstrated SVR35 in 63.2% of patients compared to 31.5% in the control arm, meeting the primary endpoint (32). These results suggest that targeting both JAK–STAT signaling and apoptotic resistance simultaneously may provide superior disease control, particularly for patients with high-risk molecular features.

In contrast to these positive results, the companion TRANSFORM-2 trial — evaluating navitoclax plus ruxolitinib in patients with relapsed or refractory myelofibrosis following prior ruxolitinib therapy — was discontinued after failing to meet its primary endpoint of improvement in total symptom score (TSS). These divergent results highlight the challenge of achieving durable symptom control with this combination in the post-JAK inhibitor setting, despite encouraging spleen volume responses in the treatment-naïve disease. Following these outcomes, the sponsor elected not to pursue further development of navitoclax in myelofibrosis.

### 5.3 Other emerging agents

Several additional agents are under investigation in combination with JAK inhibitors or as monotherapies. Luspatercept, an erythroid maturation agent, has been studied for MF-associated anemia, with mixed results regarding transfusion independence but ongoing interest for specific anemic subgroups (33). Anti-mutant CALR monoclonal antibodies (e.g., INCA033989) represent a novel immunotherapeutic approach targeting the *CALR*-mutant clone specifically, with preliminary data demonstrating improvements in symptoms, spleen size, and anemia with minimal off-target toxicity (34). PIM kinase inhibitors (e.g., nuvisertib/TP-3654), selinexor (an XPO1 inhibitor), and interferon-alpha combinations with JAK inhibitors are also under active investigation (35, 36).

The latter combination represents a pathophysiologically appealing strategy, as JAK inhibitors provide spleen and symptom control through cytokine suppression, while IFN-α exerts direct antiproliferative effects on the malignant hematopoietic clone and has been shown to reduce JAK2 variant allele frequency — an endpoint rarely achieved by JAK inhibitor monotherapy (37). The FEDORA trial is a prospective, multicenter, open-label, Bayesian phase II study evaluating the combination of fedratinib (400 mg orally, once daily) with ropeginterferon alfa-2b in JAK2 V617F-mutated treatment-naïve MF patients requiring therapy (38). Preliminary results presented at the 2025 ASH Annual Meeting demonstrated that the trial met its primary tolerability endpoint, with 87% of patients sustaining the combination for at least four months; symptom score responses, spleen volume reductions, and encouraging reductions in JAK2 allele burden were observed in a proportion of patients (39). Although FEDORA is a phase II tolerability study and was not powered for efficacy, these data provide a proof-of-concept rationale for fedratinib–IFN combinations as a potential disease-modifying strategy in MF, particularly in patients with high JAK2 allele burden where deeper molecular responses may be achievable.

## 6 Conventional pharmacotherapy

Before the JAK inhibitor era, conventional agents were the mainstay of MF treatment and remain relevant for specific clinical scenarios, particularly in lower-risk disease or as adjuncts to JAK inhibitor therapy.

Hydroxyurea remains the most commonly utilized cytoreductive agent for patients with hyperproliferative manifestations (leukocytosis, thrombocytosis) who are not candidates for JAK inhibitors or as bridging therapy. While effective at controlling peripheral blood counts, hydroxyurea has limited capacity to substantially reduce spleen volume or reverse bone marrow fibrosis, and long-term use is associated with mucocutaneous toxicity including leg ulcers (1, 40).

Recombinant interferon-alpha (rIFN-α) and its pegylated formulations (peginterferon alfa-2a, ropeginterferon alfa-2b) have demonstrated the ability to induce hematologic and molecular responses, particularly in early-stage or low-risk MF. Ropeginterferon alfa-2b, currently approved for PV, is increasingly utilized in early MF due to its disease-modifying potential and capacity to reduce the malignant clone burden, potentially delaying disease progression (37).

For patients with symptomatic anemia and low serum erythropoietin levels (<500 mU/mL), erythropoiesis-stimulating agents (ESAs) can provide benefit. Danazol, an attenuated androgen, is recommended by NCCN guidelines for anemia management in MF, with response rates of approximately 30% (3). Thalidomide and lenalidomide, often in combination with prednisone, have shown modest efficacy for anemia and splenomegaly in select patients, though their use has largely been supplanted by JAK inhibitors (1).

## 7 JAK inhibitors in the peri-transplant setting

Allogeneic hematopoietic stem cell transplantation (allo-HSCT) remains the only curative therapy for MF. JAK inhibitors, particularly ruxolitinib, are now increasingly integrated into the transplant algorithm, serving dual purposes: bridging patients to transplantation by improving performance status and reducing spleen volume, and managing post-transplant complications including graft-versus-host disease (GVHD).

### 7.1 Pre-transplant bridging

Patients with massive splenomegaly frequently experience delayed hematopoietic recovery and graft failure following allo-HSCT. The reduction of splenic volume achieved by JAK inhibition may improve engraftment kinetics and reduce the need for pre-transplant splenectomy, a procedure carrying significant operative risk in MF (41, 42). Clinical data suggest that patients who achieve a clinical response to ruxolitinib prior to transplant exhibit superior post-transplant outcomes compared to those who proceed to transplant after losing response or experiencing drug failure (43). Current consensus therefore favors proceeding to allo-HSCT during the phase of maximal response to JAK inhibitor therapy rather than delaying until disease progression (44).

### 7.2 Discontinuation syndrome and tapering

A critical peri-transplant consideration is the risk of ruxolitinib discontinuation syndrome (RDS). Chronic JAK inhibition leads to compensatory upregulation of inflammatory cytokines, and abrupt cessation can precipitate a cytokine storm characterized by acute splenomegaly rebound, fever, respiratory distress, and hemodynamic instability (43). Tapering strategies are essential: gradual dose reductions over days to weeks prior to conditioning are generally recommended (42). More recent evidence suggests that continuing ruxolitinib through the conditioning phase until stable engraftment is feasible and may prevent inflammatory rebound, though this approach requires careful monitoring of the interplay between residual JAK inhibition and conditioning chemotherapy (44).

### 7.3 Impact on engraftment and GVHD

The impact of peri-transplant JAK inhibition on engraftment remains under investigation. While there are theoretical concerns that JAK inhibitors could impair stem cell homing and proliferation, analyses indicate that with appropriate tapering or maintenance strategies, primary graft failure rates are not significantly increased (43). The reduction in inflammatory cytokines and splenic sequestration may counterbalance myelosuppressive effects, though vigilance for poor graft function and early post-transplant cytopenias is warranted (42).

Beyond bridging, JAK inhibitors have an established role in managing GVHD. The pathophysiology of GVHD relies heavily on JAK–STAT signaling, which mediates donor T-cell activation and pro-inflammatory cytokine production. Ruxolitinib has demonstrated high efficacy in steroid-refractory acute and chronic GVHD, leading to its regulatory approval for these indications (45). By suppressing donor T-cell expansion while preserving regulatory T-cells, ruxolitinib facilitates immune tolerance. Emerging data suggest that continuation of ruxolitinib post-transplant may reduce severe GVHD incidence and potentially decrease relapse risk by modulating the pro-inflammatory milieu that supports malignant clone survival (44).

As the pharmacological landscape evolves, agents such as pacritinib may serve as attractive bridging options for thrombocytopenic transplant candidates owing to its lower myelosuppressive potential. However, ruxolitinib remains the standard with the most extensive safety data in the peri-transplant setting (45).

## 8 Non-pharmacologic interventions

### 8.1 Splenectomy

Non-pharmacologic interventions for splenomegaly in MF are summarized in **Table 4**. Splenectomy provides immediate mechanical decompression and can improve cytopenias by eliminating splenic sequestration. However, splenectomy in the MF setting carries substantially higher risk than in benign indications. Perioperative morbidity rates of 25–30% and mortality rates of 5–10% have been reported, with major complications including abdominal vein thrombosis (portal/splenic vein thrombosis), post-splenectomy leukocytosis and thrombocytosis, overwhelming post-splenectomy infection (OPSI), and hemorrhage (1, 46, 47).

**Table 4:** Non-pharmacologic interventions for splenomegaly in myelofibrosis.

Procedure	Advantages	Disadvantages / Limitations	Role in MF
Splenectomy	Immediate mechanical decompression; resolves sequestration-related cytopenias; definitive treatment for refractory symptoms	High morbidity (25–30%) and mortality (5–10%) in MF; OPSI risk; post-splenectomy thrombocytosis and portal vein thrombosis; accelerated hepatic EMH	Last-resort for drug-refractory massive splenomegaly; bridge to transplant in select cases; experienced centers only
Partial Splenic Embolization	Minimally invasive and repeatable; reduces spleen volume while preserving partial immune function; avoids major surgery	Post-embolization syndrome (pain, fever); risk of splenic abscess, pleural effusion, portal vein thrombosis (increases if >70% embolized)	Alternative for poor surgical candidates; bridge to allo-HSCT; palliative for refractory sequestration
Splenic Irradiation	Non-invasive; reduces spleen size in ~72% of cases; relieves pain in ~59%; useful pre-transplant (97% spleen reduction)	Transient effects (6–12 months); risk of severe prolonged cytopenias (18.5%); not disease-modifying	Palliative for non-surgical/non-pharmacologic candidates; pre-transplant cytoreduction
Radiofrequency Ablation	Minimally invasive; preserves partial splenic function; low complication rate; short recovery	Limited hematologic improvement; may require repeat procedures; very limited MF-specific data	Investigational in MF; may be considered in select non-surgical patients at specialized centers

allo-HSCT, allogeneic hematopoietic stem cell transplantation; EMH, extramedullary hematopoiesis; MF, myelofibrosis; OPSI, overwhelming post-splenectomy infection.

Historically, splenectomy was performed more frequently for refractory splenomegaly. With the advent of JAK inhibitors, its role has narrowed to carefully selected patients who are refractory, intolerant, or ineligible for JAK inhibitor therapy and clinical trials, and who have massive debilitating splenomegaly (1). Splenectomy should be performed at experienced centers, with meticulous perioperative management including vaccination against encapsulated organisms and antimicrobial prophylaxis (46).

Partial splenectomy (PS) may offer a more favorable risk profile by minimizing complications while retaining splenic immune function. Research suggests that preserving 25–30% of splenic tissue is sufficient to maintain adequate immune protection and reduce the risk of OPSI (48). However, data specific to MF are limited, and the benefits of PS may be temporary due to splenic remnant regrowth.

The role of pre-transplant splenectomy remains controversial: some data suggest faster count recovery but potential relapse trade-offs, while other series indicate survival benefit without increased relapse (1).

### 8.2 Partial splenic artery embolization

Partial splenic embolization (PSE) is a minimally invasive procedure that induces controlled partial infarction of the spleen (typically 50–70% of splenic volume) through catheter-directed delivery of absorbable gelatin sponge particles via the femoral artery. PSE can be considered an alternative to splenectomy for patients who are poor surgical candidates due to coagulopathy, frailty, or prohibitive operative risk (1, 49).

Complications include post-embolization syndrome (fever, left upper quadrant pain, and nausea), which is nearly universal, as well as less common but serious events such as splenic abscess formation, large pleural effusions, portal vein thrombosis, and pneumonia. The severity of complications increases with the volume of splenic tissue embolized, with the highest complication rates observed when more than 70% of the spleen is infarcted (49, 50). The optimal degree of embolization for MF-related hypersplenism has not been established; insufficient embolization may fail to improve cytopenias, while excessive embolization increases abscess risk.

Evidence specific to MF is limited, but small case series suggest that PSE can achieve platelet count improvements and serve as a bridge to allo-HSCT by reducing tumor burden before conditioning, or as a palliative measure for refractory thrombocytopenia caused by splenic sequestration (1).

### 8.3 Splenic radiofrequency ablation

Splenic radiofrequency ablation (RFA) is a minimally invasive technique that employs radiofrequency energy to thermally destroy a targeted portion of splenic tissue through percutaneously inserted needle electrodes under ultrasound guidance. This approach replicates the effects of partial splenectomy while preserving normal splenic function, as retention of at least 25% of splenic tissue is sufficient for adequate immune function (51).

RFA has been applied primarily for hypersplenism in the setting of liver cirrhosis, benign splenic lesions, and metastatic disease (51–53). Its use in MF specifically is not well established, but it may be considered in select non-surgical patients at specialized centers. The principal adverse effects include mild abdominal pain, referred left shoulder pain, minor post-procedural ascites, and asymptomatic hydrothorax (51, 52). Compared to surgical splenectomy, RFA is associated with lower morbidity and mortality but provides more modest hematologic improvements and may require repeat procedures.

### 8.4 Splenic irradiation

Splenic irradiation (SI) serves as a palliative option for symptomatic splenomegaly, targeting pain relief, early satiety, and cytopenias due to sequestration. It is predominantly used for hematologic malignancies, most frequently MPNs and chronic lymphocytic leukemia (54).

A systematic review and meta-analysis by Zaorsky et al. encompassing 486 patients demonstrated that SI reduced spleen size in 72% of treatment courses, alleviated splenic pain in 59%, and improved cytopenias in 78% of cases. However, the majority of responses were partial, with symptom relief typically lasting 6–12 months. Patients with high transfusion requirements or advanced disease exhibited poorer responses. Significant adverse events, primarily cytopenias, were reported in 18.5% of treatment courses (54).

In the pre-transplant setting, a global collaborative study by Gagelmann et al. involving 59 MF patients who received SI prior to allo-HSCT demonstrated a significant and rapid reduction in spleen size in 97% of patients, with an associated lower relapse rate post-transplant. Thrombocytopenia was the most common adverse event (55). SI may therefore serve a valuable role as a bridge to transplant, particularly when JAK inhibitors are insufficient for spleen volume reduction.

SI is a palliative measure and not a definitive treatment; its effects are transient, and the risk of prolonged severe cytopenias must be carefully weighed against the expected benefits.

## 9 Future directions and unmet needs

Several critical gaps remain in the management of MF-related splenomegaly. Earlier intervention strategies to prevent the development of massive splenomegaly are needed, as is the identification of biomarkers predictive of spleen response and resistance to JAK inhibitors. Therapies capable of eradicating the malignant stem cell clone and truly modifying the disease course remain an aspirational goal (1). A consensus definition of “disease modification” is needed to guide the design of clinical trials with endpoints beyond SVR35 and TSS, incorporating bone marrow fibrosis regression, variant allele frequency reduction, and overall survival (35). The integration of combination strategies with transplant timing and the evaluation of novel agents in the peri-transplant setting represent active and important frontiers.

## 10 Conclusions

Splenomegaly is a defining and morbid feature of MF that drives symptoms, cytopenias, and vascular and hepatic complications. JAK inhibitors constitute the backbone of spleen and symptom management and have transformed patient care over the past decade. Ruxolitinib and fedratinib remain central for many patients, while pacritinib and momelotinib have expanded the therapeutic options to encompass patients with severe thrombocytopenia and anemia-dominant disease, respectively. The advent of combination therapies—particularly pelabresib and navitoclax with ruxolitinib—represents the next major advance, with the potential to achieve deeper and more durable responses. Sequencing strategies after JAK inhibitor failure, incorporation of clinical trials, and selective use of non-pharmacologic procedures (splenectomy, splenic irradiation, PSE, and RFA) remain essential components of comprehensive MF splenomegaly management. A phenotype-driven, individualized approach to therapy selection, guided by the patient’s cytopenia profile, symptom burden, transplant candidacy, and available clinical trials, offers the best prospect for optimizing outcomes.
